# Good cancer follow-up for socially disadvantaged patients in general practice? Perspectives from patients and general practitioners

**DOI:** 10.1080/02813432.2024.2317843

**Published:** 2024-02-20

**Authors:** Lotte Lykke Larsen, Camilla Hoffmann Merrild

**Affiliations:** Center for General Practice at Aalborg University, Aalborg, Denmark

**Keywords:** Socially disadvantaged, cancer care, general practice, nordic individualism, qualitative

## Abstract

One of the core principles of providing care in general practice is giving more to those who need it most. We investigate some of the complexities of this ambition in the context of cancer care for patients defined as socially disadvantaged by their general practitioner (GP). We do this by exploring how care is sought, how it is offered, and what expectations patients and GPs carry with them when receiving and providing cancer care in the Danish welfare state. We carried out semi-structured interviews with eight GPs and seven socially disadvantaged cancer patients living with different types and stages of cancer. The interviews focused on needs and challenges in cancer follow-up in general practice and were thematically coded. Drawing on theoretical concepts of morality and Nordic individualism, we point to how one of the main challenges in cancer care and follow-up is to figure out how the doctor-patient relationship should be established, practiced, and maintained. Both GPs and patients stressed the importance of the relationship, but how it should be practiced amidst social norms about being a patient, a citizen and how care-seeking should unfold seems less clear. In conclusion we argue that giving more to those who need it the most is a difficult and ill-defined task that is shaped by the cultural, social, and political expectations of both GPs and patients.

## Introduction

Globally, more people survive being diagnosed with cancer and the increase in survival has led some to consider cancer as a chronic condition [1]. In Denmark, the increase in cancer survival has been attributed to the effectiveness of the cancer care pathways, which ensure a fast-track process from the first presentation of alarm symptoms to diagnosis and treatment [2]. The follow-up cancer care programmes were reformed in 2015, with general practice given increased responsibility for caring for cancer patients in uncomplicated and curative disease trajectories [3]. When routine, hospital follow-up is terminated, or reduced in intensity, cancer patients are primarily affiliated with general practice rather than the specialised departments where they received their diagnosis, treatment and, sometimes, cure. Thus, general practitioners (GPs) are identified as coordinators (*in Danish: tovholder*) in caring for uncomplicated cancer patients, who join many other patients with chronic illness who have finished hospital treatment, live with their disease, and are cared for in the context of general practice [4]. One of the challenges involved in this transfer of scheduled follow-up care and support from the specialist secondary sector to more ad hoc care in the primary sector, has been the lack of cooperation between sectors. This was identified in a review of GPs’ role in cancer follow-up [5], and supported by a recent report on cancer follow-up and palliation in Danish General Practice [6]. However, the review also showed that across all studies patients were positive towards the idea that their GP played a greater role in cancer care [5], underlining the value of the close relationship between patients and their GPs and accessibility to general practice services. The Danish report also noted that most GPs felt confident that they were able to care for their cancer patients [6].

By locating cancer care for uncomplicated patients in general practice, attention to potential relapse and late effects depends on patient care-seeking practices, as GPs do not usually carry out systematic cancer follow-up. This requires bodily attention and vigilance [7], which may be challenging for cancer patients who also live in socially disadvantaged situations [8]. Recent studies have shown social differences in relapse [9], and there are significant social differences in how people with different social backgrounds fare after being diagnosed with cancer [10,11]. Studies have shown social differences in rehabilitation, multimorbidity, and quality of life [12–14]. People living with cancer are more likely to be affected by other chronic disorders than those who have not had cancer, a tendency which increases with social disadvantage [11]. One implication of this may be that sorting through signs of cancer and other illnesses presents challenges when living with multiple social and medical problems [15]. Thus, it seems reasonable to suggest that the move away from scheduled cancer follow-up, towards more general and self-initiated care in the primary sector can be challenging for socially disadvantaged cancer patients, as it assumes proactivity, vigilance, attention to bodily changes, and subjective needs for care. In previous studies with socially disadvantaged cancer patients, it has been pointed out that experiences of bodies and care-seeking practices are shaped by overall health status, pragmatism, and instability, largely influenced by the practicality of getting through the day [8, 16].

Danish general practice is organized around principles of free and equal access for all citizens. One of the seven key indicators in Danish general practice is to ‘give more to those who need it most’ [17], an approach to offering proactive care which resonates with the core Values and Principles of Nordic General Practice, as formulated by the Nordic Federation of General Practice [18]. This approach is needed as patients who live in socially disadvantaged situations are more likely to have unmet needs in their cancer rehabilitation [19]. How it should be practiced, however, is less clear, despite being a key principle in the guideline on care for cancer patients [4]. In this study we want to look more closely at how the ambition, from the key indicators, of giving more to those most in need is experienced by patients with different stages and types of cancer, who are defined by their GPs as socially disadvantaged and, conversely, how GPs perceive their role in relation to offering support to this group of patients.

## Method

The study was designed with reference to the COREQ reporting guidelines [20]. We carried out individual, semi-structured interviews with eight GPs and seven socially disadvantaged cancer patients living in different socially disadvantaged situations. The interviews were conducted as part of an intervention study with the aim of developing and testing a supportive model to improve cancer follow-up in general practice, focusing on supporting socially disadvantaged cancer patients during and after cancer treatment. The participating GPs and patients were recruited using a combination of purposive and maximal variation sampling strategy [21,22], which was applied to include GPs with extensive knowledge and experience with the needs and challenges of cancer follow-up in the context of general practice, and to ensure variation in terms of geography, practice type, experience as a GP, gender, and age [22]. The GPs were invited to participate through an advertisement in a newsletter from *the Quality Unit for General Practice in the North Denmark Region* (Nord-KAP), and through personal emails sent to GPs who had a special interest in socially disadvantaged patients and cancer (see Table 1 for an overview of the participating GPs). The inclusion criterion was motivation to participate in the whole intervention project, which would involve individual interviews, a workshop, testing the supportive model in their own practice, and evaluation of the model. In addition, the participating GPs committed to help recruit patients to the project from their own patient population. All patients were recruited by the participating GPs. The inclusion criteria for patients were a current or former cancer disease and living in a socially disadvantaged situation as defined by their GP. The GPs received information about recruitment of patients which stressed that the meaning of ‘socially disadvantaged’ was not only defined by the cancer disease but could be defined by factors such as: education, social network, civil status, connection to the labour market, selfcare, and disease insight. The GPs discussed patient selection with the first author and the diversity of this category was openly considered, both as an empirical/practical problem, but also as a conceptual problem carrying the risk of both stigmatization and marginalisation. GPs chose not to inform the participants that they found them socially disadvantaged, but during the interviews it was made clear that the focus was on how they managed life with cancer along with all other challenges they faced in daily life. All patients had given consent for their contact information to be passed to the first author, who then contacted them by telephone and arranged a time and a place for an interview. Eleven patients were recruited; two patients were too ill to participate, and two could not be reached to make an appointment, leaving seven patients interested and able to participate in the interviews. The patients had different forms and stages of cancer. By the time of the interview, none of them had been declared cured, and five out of seven patients had incurable cancer. The patients were defined as socially disadvantaged by their GPs because of factors such as multimorbidity, limited or negative social network, alcohol abuse, social challenges in the family, weak disease insight, and psychological vulnerability. See Table 2 for an overview of the participating patients.

**Table 1.** Overview of GPs.

Gender	Pseudonym	Age (interval)	Seniority as a GP	Practice type^1^	Location
Male	Peter	50–65 years	More than 10 years	Single-handed	Rural area
Male	Michael	40–50 years	More than 5 years	Partnership	Rural area
Male	Robert	40–50 years	More than 5 years	Partnership	Rural area
Female	Mia	40–50 years	More than 10 years	Single-handed	Urban area
Female	Sarah	40–50 years	More than 5 years	Partnership	Urban area
Female	Grete	50–65 years	More than 20 years	Single-handed	Urban area
Female	Charlotte	40–50 years	More than 5 years	Partnership	Urban area
Male	Thomas	40–50 years	More than 5 years	Partnership	Rural area

**Table 2.** Overview of patients.

Gender	Pseudonym	Age (interval)	Health status	Social situation	Occupation / job title
Male	Tom	80–90 years	Incurable cancer. Mental illness.	Living with his wife. Lack of disease insight.	Pensioner / engineer
Female	Linda	70–80 years	Multiple cancers, widely spread. Incurable. A lot of pain. Poor functional level.	Living alone. Limited social network.	Pensioner. Service industry
Male	John	70–80 years	Incurable cancer. COPD. Blood clot in the brain. Cognitive challenges.	Living with his wife. Alcohol abuse.	Pensioner. Farming, harbour industry
Female	Jane	60–70 years	Multiple concurrent cancers. Incurable. COPD and diabetes. Mental illness.	Newly divorced. Living alone. Unstable and limited social network. Alcohol abuse.	Early retirement before cancer. Fish and iron industry
Female	Susan	60–70 years	Multiple cancers. Curative. Several chronic conditions.	Living with her husband. Little social network, family troubles.	Early retirement before cancer. Stores and domestic help
Female	Lilly	70–80 years	Cancer. Newly operated, expects to be cured. Numerous physical late effects.	Living with her husband. Family troubles.	Pensioner. Service industry
Female	Jonna	60–70 years	Multiple cancers. Several chronic conditions.	Living with her husband, who also has several chronic conditions.	Early retirement because of cancer. Social and service industry

Interviews with GP’s took place in the period from October 2021 to January 2022 at the GPs’ clinics. They lasted between 30 and 75 min with an average of 50 min. The interview guide focused on the organisation of cancer follow-up in general practice, consultations with socially disadvantaged cancer patients, and general practice’s role in the cancer course and follow-up, all focusing on needs, challenges, and what can be improved in cancer follow-up for socially disadvantaged patients. The interviews with patients took place in their homes in the period from November 2021 to February 2022. The interviews lasted between 45 and 70 min with an average of 55 min. The interview guide centred on social network, the cancer trajectory, GPs and general practice’s role in the cancer course and follow-up, support from the healthcare system overall, with a focus on needs, challenges, and the role of general practice in cancer care. Both interview guides were developed in a collaboration between the two authors and were based on existing evidence about cancer follow-up in general practice, support for socially disadvantaged groups, and patients’ experiences in the healthcare system. Interviews were audio-recorded and transcribed verbatim. One patient, however, was not comfortable with being recorded and notes were taken during the interview instead, and the data handled like a transcribed interview afterwards. The transcripts were read several times, and a thematic coding was carried out [21]. The interviews of GPs and patients were coded separately. Coding was created in Nvivo12. The analysis followed an inductive approach, with themes developed from the data.

In the thematic coding, initial codes were made through the entire text. Afterwards overlaps were merged, and codes subsequently combined into themes. After re-reading and working on condensation of the themes we ended up with six themes from analysing GP interview data, and six themes from patient interview data. A final condensation was made, to combine the results from GP and patient interviews. The themes from the separated thematic coding were merged into three overarching subjects, where GP and patient perspectives were combined. The overarching subjects were *Support in cancer care*, *An outsider in the cancer trajectory*, and *Challenges of everyday life*.

## Ethics

All participants gave their written consent to participate. They received both written and verbal information about the project and were informed that they could withdraw their participation at any time. Patients were also assured that their relationship with their GP would not be impacted in any way by their participation or potential withdrawal from the study. All participants are anonymized in the transcribed data, and all participants were assured that other participants (e.g. own GP or patient) would only get insights to anonymized quotes, which would not affect their patient-doctor relationship. The first author was aware of the patients’ reactions and feelings during the interviews, as they were talking about emotional topics, and assurances were given that patients should only talk about what they were comfortable with.

In the presentation of the results below we draw on examples from cases combined and compared with quotes. Detailed information about the participants and any people they describe is omitted or altered in ways that have no bearing on results. The cases and quotes are selected to illustrate the content of the overarching themes from the thematic analysis. The quotes are translated from Danish into English.

## Results

### Support IN cancer care

Lilly describes herself as an ‘alone-girl’, even though she lives together with her husband. Her life has been tough, and her mother raised her to take care of herself, making it very clear that no one else will. Lilly realises the truth of this as both her own father and her previous husband and the father of her children have been unsupportive in their family life. Lilly is suffering from late effects of cancer treatment, such as being tired and lightheaded, and her current husband never joined her on her many visits to hospital. She feels that he does not take her illness seriously and he kind of withdraws by not wanting to talk about it. When asked who she turns to if she needs support or someone to talk with, she replies:

Lilly: I don’t know, I don’t think I have a great network, I don’t think so.

First author: Who do you talk to about your illness?

Lilly: (sigh) Well, he (nods towards the room where her husband is) doesn’t want to talk about it, so it’s probably mostly my daughter-in-law (…).

First author: Do you need someone to talk to about it?

Lilly: No. But I guess I probably…. I mostly just deal with it myself.

This feeling of being alone in managing the disease was common among the patient participants. They handled almost everything about their disease by themselves, and rarely talked about cancer care and late effects with their relations. Some of the patients we spoke with used their GP as someone to talk to about their cancer, whereas others, like Lilly, did not consider that as a possibility. She explains:

I don’t think my GP has time for that. I feel like, you know what, they’re so busy, I shouldn’t bother with silly stuff like that. Then I just have to puzzle with that by myself and try to get things sorted out.

Those who did choose to involve GPs in their cancer care, often did so because they found their GP to be one of the few people who understood what they were going through, as was the case with Jonna who lives in a small house with her husband. When the first author visits her, Jonna looks tired and moves slowly through the house while apologising for the smell of cigarettes, and the level of cleaning. Jonna has been on early retirement for more than 20 years due to one of the chronic cancer conditions she struggles with alongside her other chronic conditions. Like Lilly, Jonna’s husband offers little support towards her cancer care. When the first author asks her where she turns when she needs someone to talk with about her cancer, Jonna sighs:

That is probably mostly my GP now. And I can also talk to my sister, one of them. But it is also sometimes a bit difficult. Because there are things which…. I would like to keep to myself too. Because…. well because …many of those, even John (her husband), who are close to me right. They can’t, they can’t understand that I feel the way I feel. They can’t see anything, physically, right?

This lack of support and involvement of family and/or friends was common among the participants in our study, who were reluctant to share their physical, social, and mental concerns and challenges, either with partners or with close relations. They primarily kept to themselves and managed on their own, sharing very little, if anything, about their cancer experience.

When asked directly about the role played by their GP in their cancer care, the patients were very reluctant to ask for help and most of them did not want to disturb the GP. Although some would have liked to talk to their GP about their cancer, all of them worried about whether their concerns and requests were important enough to disturb their GP with, both regarding emotional topics but also in relation to physical discomfort and late effects. As participant Jane explains: ‘I often think, well, what you come up with is probably just nonsense. Even though it is important to me (…) I don’t know, I mean, I could just say something, but I don’t’.

This perspective of *not wanting to be in the way* was not lost on the GPs, who wanted to be more proactive towards socially disadvantaged patients, who they described as patients who rarely ask for help and who express their needs less than more socially advantaged patients. The GPs all referred to the importance of helping those who were most in need, typically followed by a narrative of the challenges of providing that help and how cancer patients’ needs differ according to cancer type, as well as from patient to patient. As was evident in their recruitment of patients, they often defined the socially disadvantaged patients as those who lacked close and supportive relations in their lives, and they were aware of the challenges of patients being alone in their cancer care. They would highlight the importance of following these patients closely, and most of the GPs considered their role in cancer care to be about building relations and ensuring continuity. Mia, who has been a GP for 12 years in a single-handed practice, said:

I can’t take care of their oncology treatment, nor can I say if it’s the right or wrong one. But I can be a coordinator for them, in relation to pain issues or anything else, where they kind of say, you, you see me over time, so you have the continuity and stuff like that (…). So, I believe that, at least this relationship matters extremely.

However, although the GPs recognised the importance of the relationship with their patients, there was a significant difference between how GPs and patients talked about it and what each of them felt constituted good care. The GPs were mostly concerned about knowing who the cancer patients were, being updated about their cancer treatment status, and thereby meeting the expectations of what they considered to be the elements of a long-term relationship with patients. They assumed that knowing the patients’ illness trajectories and when to proactively approach those who needed close care and support would be a good thing. Patients, on the other hand, were primarily interested in the immediate doctor-patient connection. Jane described it like this:

This doctor he is like, when you come into the consultation room, he just looks at the screen and then he turns around and talks with YOU. Many of them, they just sit and look at the screen, and you just feel like something that is just there. But my GP he doesn’t have to, he has read it all before you come into the room. He is just so human.

The patients wanted the GPs to be precise and direct when they talked about cancer, but also to provide comfort, which was mentioned as one of the most important tasks in cancer care in general practice. They valued simple things like the GP being present, caring, and listening in the consultation.

### An outsider in the cancer trajectory

The challenges for GPs of keeping informed about patients’ cancer trajectories were described primarily at the organisational level and in aspects of cross-sectoral cooperation. Patients with complicated trajectories and multiple disease who were followed by hospital departments were the most difficult patients to keep track of. Robert is a GP in a newly established partnership practice in a small town which has been underserved by GPs for years. Many of his patients have had multiple, short-term GPs, and he describes the difficulties involved in keeping track of patients’ cancer care:

We may follow them in the beginning when they get their cancer diagnosis through the discharge summaries. But then it’s as if it gets a little muddled by the amount of information we receive and we don’t quite know when the care at the hospital ended, and when they were actually discharged, and when they move on to be an outpatient. In that way they probably fade out a bit. And then it often happens that the patient shows up here half a year later and says, And by the way, I had cancer’, and then we are like, Oh God, yes. That’s true, there was a cancer trajectory there. So, it will probably, unfortunately, take us a bit aback. (…) It would seem cool right, if I had known when he came through the door, or perhaps on top of that I had contacted him along the way.

Patients also pointed out that communication and handing over information across sectors is important. They found cancer follow-up in general practice difficult and somewhat out of place, as their GP had not previously been part of their cancer care. None of the patients we spoke with requested cancer follow-up in general practice, although it did make them feel more comfortable if their GP was updated about their cancer treatment status. They underlined how this would improve the relationship with their GP, and as Jonna put it:

It would be a good thing in relation to your doctor, where you feel that they care about you, or take care of you if needed. And if they could just call and say they have received the message and ask how you are doing and, stuff like that.

The challenges of maintaining an overview and keeping track of new cancer patients was evident in how GPs described forgetting or not knowing about patients’ cancer diagnoses or care trajectories as an embarrassment. Not having accurate information to hand made it difficult for GPs to be involved in their patients’ cancer care and sometimes patients ended up carrying information between sectors themselves. Sarah, a GP in a partnership practice, describes a missing communication between the primary and secondary sector:

What can be difficult, sometimes, and what I might miss, is when a patient goes from receiving curative treatment to, there is dissemination, we quit, and this is now a palliative treatment. THIS IS WHEN I miss contact from the hospital. (…) And this is where I would like some kind of agreement to be made, that the family doctor must be informed about it. Because treatment is still ongoing, but they do become a mentally fragile human the moment you are told that your prognosis has changed.

Communication between the sectors was often confined to discharge summaries and GPs receive many of these every day, which may cause some information to be overlooked. The lack of information sharing across sectors sometimes resulted in missed communication between patients and their GP, but also in missed opportunities for the GP to support their patients, particularly when patients are in the palliative stage of cancer care.

### Challenges of everyday life

‘Back then, when I got sick there in, well, it was probably in 67, I think, eh… No, when I was 67, I thought that nobody would bother spending money on me, I was absolutely sure about that’.

John is 74 years old, and the first author meets him at his small farm, where he lives with his wife. John looks tired and his body is worn out from a lifetime working on the farm and in the harbour industry. During his first cancer treatment he was on a respirator, and he barely survived, he tells the first author as they sit down by the sofa table in their conservatory. The first author asks him how he would describe his overall health: ‘Well. I suffer like hell with that COPD, otherwise I think I’m fine’. John has just recently been informed that his cancer is incurable, and that the current treatment is palliative. Nevertheless, he is feeling well, and cancer worries do not seem to figure prominently in his everyday life, except for some matter-of-fact practical considerations that he wants to sort out before he dies.

In line with John’s story and perspective, GPs often recognised that their role in cancer care for socially disadvantaged patients was about helping them with practical matters. Thomas, a GP in a partnership practice in a rural area said: ‘Sometimes it is just solving the trivial, practical, troublesome (…) it is mostly, being able to talk to the patients about what is important to them’.

Reaching out and helping socially disadvantaged cancer patients, however, is not always straightforward, partly due to the patients’ uncertainty about what to expect from the healthcare system. This matches the GPs’ concerns that some of their socially disadvantaged patients do not get the support they need, as they do not ask for anything. When asked about their need for assistance and support the patients did indeed refer to very practical things that would ease their daily life, such as support stockings, a walker, or a motorised wheelchair to help them get around; or they would mention support from friends and relatives in managing daily tasks. But mostly they stated that they managed on their own, and none of them referred to anything they needed help with that related to their cancer disease or symptoms. Many of the patients have multiple chronic diseases, and cancer is often not the disease that affects and restricts them most in their everyday lives. As John made clear earlier in this section, cancer did not dominate his feelings or his perspective on his overall health. Likewise, when Jane, who also has COPD, was asked how her diseases affect her life she said: ‘Well – this lung thing is annoying… Because cancer, well that, I don’t think much about that because it’s not what bothers me. Well, there are those after-effects. But otherwise, the cancer itself, I don’t think much about that’. This is also explained by Jonna, who tells what bothers her the most in everyday life:

Well, what matters most, is the fact that I am not as capable as I used to be. I wish that I could have a little more energy, get started with things. Be able to finish things. I almost never get there. And finishing things and that, that’s hard for me. To accept that. But I guess I have to.

Cancer was not a big part of the patients’ everyday lives. They had been diagnosed, treated, and most of them were living with incurable, chronic cancer. That is not to say they were not confined by their cancer; practical tasks have become harder, take more energy, and energy is sparse:

## Discussion

### Cancer care, the welfare state, and Nordic individualism

During our interviews the challenges of being alone in cancer follow-up surfaced again and again alongside uncertainties about how and when to ask for help. Our interlocutors did not want to burden their GP with unnecessary concerns, a common narrative among Danish citizens [23]. However, hesitancy and not asking for anything may also be considered an aspect of the moral contract between the state and the citizen, which was first alluded to more than 30 years ago by Norwegian anthropologist Marianne (24], as a form of Nordic individualism where managing on one’s own and being independent are highly valued even when living in an extensive welfare state. This moral responsibility of managing on their own also has similarities with a recent study of Danish cancer patients living alone [25], which shows that being dependent on family and friends when ill can be experienced as a moral dilemma and a kind of exchange that may compromise relationships. For instance, the authors point to the importance of maintaining a sense of normality in family life, as well as avoiding burdening other people with their problems, and they illustrate how different aspects of care, such as physical and bodily care is considered to belong to the professional caretaking. This suggests that the provision of health and home care is grounded in contrasting political and moral expectations of the welfare state, and in classifications of when and what care belongs to the professional and personal sphere. This lead the authors argue that some contradictions are implicit in the social contract between Danish citizens and the welfare state that are bound up with morality, family relationships, welfare politics, and the practicalities of everyday life when living alone following cancer treatment [25]. Like the participants in Andersen and Offersen’s study, our interlocutors did not want to rely on friends or relatives, and they were reluctant to ask the GP for help. The GPs, on the other hand, recognized the reluctance but were insecure about how to provide care that was not directly asked for. However, being aware and attentive towards the patients was a significant challenge, particularly in relation to the transfer of responsibility for care. The troubles our GPs describe with lack of information and being ‘disconnected’ from their patients’ cancer care, resonate with other studies [26,27], and insufficient communication and coordination between primary and secondary care has been identified as a barrier for the involvement of general practice in cancer survivorship care [28]. A systematic review also highlighted that when GPs felt disconnected from patient care, they were unsure about when to re-establish contact with the patient [5, 29].

Another key point that came out during our interviews was how the struggle to manage the challenges of everyday life when living in socially disadvantaged situations seemed to overshadow the challenges of living with cancer. This finding shows similarities with a recent systematic mixed studies review, which points to how prioritisation by patients is closely tied with their illness experiences, whereas clinicians focused on longer-term risks and challenges [30] Other studies focusing on patients with a cancer diagnosis and comorbidities also found that people pay attention to symptoms that affect their everyday living and function, rather than their disease [16, 31]. However, one very important point raised by [32] is that when cancer patients say that they are doing fine, this does not necessarily correspond with the pain and suffering that they live with in daily life. The authors suggest that *being OK* for people with advanced cancer bears significantly different connotations than what might be considered as OK according to the norms of a person who is well and healthy. Certainly, these findings highlight the need for a nuanced understanding of the experience of *doing well* under circumstances of severe illness [32, 132]. One point we wish to add relates to uncertainty about what to expect from the healthcare system. Researchers looking at the historical development of the Danish welfare state have argued that welfare benefits increasingly seem to come with a set of expectations rooted in ideas of managing on one’s own, referring to post-social reform Danish society as the independence society [33]. The reluctance to seek care from general practice may reflect a moral obligation of only using the healthcare system when it is necessary [23], but it also carries a sense of not being good or deserving enough [34]. In Nordic contexts it has been argued that individual autonomy is institutionalised through a range of welfare initiatives, ‘*through a plethora of laws and policies affecting Nordics in matters minute and mundane as well as large and dramatic*’ [35, 15]. For cancer patients, follow up in general practice is one example of how this works in practice. On the one hand, the GP is positioned as care coordinator maintaining an overview of the patient’s cancer trajectory when they are discharged from specialist treatment; while on the other, patients must actively seek care and support when they need it, which makes initiative and proactivity the personal responsibility of the patient. This, our study shows, is an ill-defined and difficult task. For those patients who live difficult and challenged lives more handheld support would seem beneficial, and perhaps an even reversed proactivity which places the responsibility on the health care system.

### Methodological discussion

The findings of this paper should be interpreted with the choice of methods in mind. First, the article draws on a sample of GPs who have volunteered to participate in a project about improving cancer care for socially disadvantaged patients, which indicates that the GPs have a special interest in the subject, and they may thus go to greater lengths to offer care and support. This, however, is a condition when using purposive sampling and while recruiting we ensured maximal variation as a sampling strategi, which meant that the GPs varied in terms of geographical setting, age, seniority, and practices type.

Allowing the GPs to recruit the patients they considered socially disadvantaged also presented a challenge. Applying a category such as socially disadvantaged may lead to recruitment based on misunderstandings and subjective assessments. However, we chose to use the categorisation socially disadvantaged rather than for instance socially vulnerable in order to stress some of the contextual factors of living in difficult life situation, while we fully acknowledge that it is a simplification of very diverse lives, as has been stressed by others working with marginalized groups [36]. Leaving the GPs in charge of selecting the patients allowed for the risk that they would not fit the descriptions or indeed need the extra support that the GP expected, and it may even be argued that socially disadvantaged is not a particularly useful category when it comes to defining those patients who need the most care and support. However, aligning the selection of participants with the realities of Danish General Practice, where the key indicator, as mentioned earlier, allocates resources to those in greatest need, we opted to allow the GPs to determine patients requiring additional support. Overall, our methodological challenges in recruitment reflects that challenges of giving more to those who need it most, particularly in defining who constitutes ‘those in need’ and by whose criteria? And perhaps just as important, our results seem to speak directly into this fundamental challenge of tackling social inequality in health.

Our method of semi structured interviews provided us with rich data in face-to-face interviews. Particularly visiting the patients in their own homes offered insights into their day-to-day life, their living situations and sometimes also their family. It also created a homely and relaxed setting for discussing sometimes difficult and emotionally topics. The interviews with the GPs were supplemented with workshops which allowed both authors to engage with the GPs and discuss their perspectives, and also provided the opportunities for the GPs to discuss challenges and solutions with each other.

## Conclusion

In this article our aim was to understand how cancer care is experienced by people who live in socially disadvantaged situations, and how GPs perceive their role and how they provide cancer care amidst these disadvantages. We point to some of the contradictions in how good care is imagined and envisioned by patients and doctors respectively, and one of our main findings was that while both patients and GPs emphasised the importance of the relational aspects of providing good care, there was a difference in how this was perceived in practice. GPs were primarily concerned about the organisational challenges of not having a sufficient overview of which patients needed most care and support. They suspected that the patients they considered as the most vulnerable often refrained from contacting them, and while they wanted to be more proactive towards this patient group, they lacked both time and an overview of the patients’ situations and cancer trajectories. This was further complicated by the fact that GPs were not always aware when patients had been discharged from hospital, and that they had thereby become responsible for follow-up in their capacity as care coordinators. Patients, on the other hand, were mostly concerned about the interpersonal and relational aspects of consultations with their GP, and it was very important to them that the GP was present and attentive in the consultation, asking directly about their cancer, and allowing space for their concerns and for providing comfort. Although most of our interlocutors dealt with their concerns about cancer alone, despite having a life partner, some did mention their GP as the only person they could talk to about their illness. Social support is important in cancer rehabilitation, and relatives play an important role in supporting patients to cope with their cancer disease and treatment [37]. Our interlocutors did not have this social support and were used to managing many aspects of life alone, which shaped their illness experiences and moral practices of care-seeking. Apart from sharing their challenges and concerns with the GP, they did not ask for care or support. They had few expectations that their GP could help or care for them in everyday life. Our participants experienced general practice as a busy place, and they were unsure about whether their concerns and struggles were important enough to bring to the attention of the GP. At the same time, patients considered cancer care to belong to specialists at the hospital, where they had followed a strictly defined, scheduled trajectory. They did not really see how general practice could or should support them in relation to their cancer disease. Meanwhile, the GP’s role was complicated by uncertainties about when responsibilities for caring for socially disadvantaged cancer patients transferred from the secondary care sector to general practice.

Patients not wanting to disturb their GP may be seen as an example of the moral obligation of responsible use of healthcare resources as pointed out by others [23]. We want to draw attention to the implications of this obligation when it comes up against the intention is to give more to those who need it most. We argue that one of the main challenges in cancer care and follow-up is to figure out what the GP-patient relationship consists of and how it may be established and maintained. The relationship is valued by both parties, but they struggle to find its form amidst cultural, political, and social norms about how to practice illness and care-seeking and what good care is really about. Although this is difficult, and fraught with uncertainty this does not mean that the ambition of giving more to those who need it the most should be abandoned. However, it should perhaps become more invested in the real lives and needs of those patients who need extra care, and tailored to the realities of what is at stake in their everyday lives, rather than how they are envisioned as socially disadvantaged.

## Data Availability

The datasets generated during and analysed during the current study are available from the corresponding author on reasonable request.

## Notes

1 Single-handed type: an individual/solo practice. Partnership type: a practice with more GPs in the same clinic, in partnership or cooperation.
